# Chloroform in Infantile Convulsions and Other Spasmodic Diseases

**Published:** 1852-10

**Authors:** 

**Affiliations:** Edinburg


### RECORD OF MEDICAL SCIENCE.

### OBSTETRICS.

*Chloroform in Infantile Convulsions and other Spasmodic Diseases.* By Professor SIMPSON, Edinburg.—As the majority of convulsive attacks in infants depend upon sympathetic or functional derangements, and not on structural changes, the first indication is to discover and remove sources of irritation; and the second, to reduce super-irritability of the excito-motory system. In the more chronic cases iron and zinc are used; in the more acute ones, antispasmodics; such as opium, hyoscyamus, and musk.

*Case.*—The Viscountess —— was confined on the 7th of October. On the 17th of the same month, the child was observed by the nurse to have two or three times during the day twitchings in the muscles of the face. On the two following days these increased in frequency and extent; on the 20th, the convulsions became far more violent in their character, were more prolonged in their duration, and were repeated with much greater frequency. They continued with little change, and no abatement in their intensity or frequency, for the next fourteen days. Sometimes they affected the right side of the body much more severely than the left. In the meantime, Dr. Scott and I tried a great variety of means for their relief, but all in vain. The bowels were well acted upon with mercurials, magnesia, &c.; and every separate function attempted to be brought as near as possible to the standard of health. A new wet nurse was procured, lest the milk might perchance have been proving, as it sometimes does, the source of irritation. The child was placed in a larger and better ventilated room. Ice and iced water were occasionally applied to the scalp. At one time, when the fits became unusually prolonged, and were not only accompanied but followed for a time by much congestion in the vessels of the scalp and face, and an elevated state of the anterior fontanelle, two leeches were applied. Liniments of different kinds were used along the spine. Musk, with alkalies, was given perseveringly for several days as an antispasmodic; and small doses of opium, turpentine enemata, &c., were exhibited with the same view. All these, and other means, however, proved entirely futile.

As I have already stated, it was on the 20th of October that the fits first assumed a severe character, and they continued without any amelioration for about fourteen days from that period, recurring sometimes as frequently as ten or twelve times in an hour. At last the child, who had hitherto maintained wonderfully his strength and power of suction, began to show symptoms of debility and sinking; and during the fifteenth and sixteenth days of the attack, the fits became still more violent, and more distressing in their character. They were now accompanied with moans and screams that were very painful to listen to; symptoms of laryngismus and dyspnœa supervened towards the termination of each fit; and in the intervals the respiration, as well as the pulse, continued much quickened.

During these two last days of the disease, the exhaustion became so great, the dyspnœa in the intervals so distressing, and the fits so very violent and constant (seventeen were counted in one hour,) that Dr. Scott and I gave up all hopes of the possible survival of the infant. We had exhausted all the usual means of relief. Ultimately, but much more with the view of abating the screaming, laryngismus, and other distressing symptoms under which the little patient was suffering, than with any great hope of permanent relief and cure, I placed the child, on the forenoon of the 5th of November, for about an hour under the influence of the inhalation of chloroform. During this hour there was no recurrence of the fits; but in a short time after the withdrawal of the action of the anæsthetic the convulsions recommenced with their old violence and frequency. The benefit, however, was sufficient to encourage a longer repetition of the remedy; and from four to eight o'clock in the afternoon of the same day, my assistant, Mr. Drummond, placed and kept the child again under the influence of chloroform, a few inhalations from time to time, of a very small quantity of the drug sprinkled upon a handherchief, and held before the face of the infant, being sufficient for this purpose. It was specially applied at any threatening of the recurrence of a fit, and during the four hours in question, all convulsions were in this way repressed. When the child was allowed to waken up at eight o'clock, it took the breast greedily, and continued well for upwards of an hour, when the convulsions again began to recur. At last, about 12 p. m., it was again placed under the inhalation of chloroform, and kept more or less perfectly under its action for upwards of twenty-four continuous hours, with the exception of being allowed to awaken eight or ten times during that period for the purpose of suction and nourishment. During most of this period it was carefully watched by Mr. Drummond, and at last the nurse was entrusted with the duty of adding the few drops of chloroform to the handkerchief, and exhibiting them at any time the child was offering to awaken or become restless.

After this long continuation of the chloroform, the child, on being allowed to waken up, as usual drank greedily at the nipple, and immediately fell back into a quiet and apparently natural sleep. The chloroform and all other formal medication was in consequence discontinued; and from this time there was subsequently no recurrence whatever of the convulsions. In about ten days the infant was removed with the family to the country. I have within the last two days (December 18,) seen the child as it was passing through Edinburg. It was strong, plump, and well grown for a child ten weeks, and was, in fact, revelling in the best of health.

In exhibiting the chloroform to this infant, ten ounces of the drug were expended; but of course a very large proportion of this quantity was lost by evaporation, in consequence of the mode in which it was employed.

I have known the inhalation of chloroform similarly useful in other cases in arresting infantile convulsions; but I am not acquainted with any instance in which the patient was so young as in the above instance. In the adult also, especially in cases of puerperal convulsions, I have now repeatedly seen the inhalation of chloroform as signal and satisfactory in its antispasmodic power over the convulsive fits, as it was in the little patient whose case I have described. Tetanus and epilepsy have been temporarily arrested and controlled by it; and perhaps it will yet be found one of our most certain and beneficial therapeutic means in the functional forms of those different convulsive or spasmodic diseases that are produced either by an undue excitability of the true spinal system, or by distant morbid irritations acting through this, the excito-motory system. Such reflex convulsive or spasmodic affections are, as is well known, particularly common in infancy and childhood. I have seen its use arrest laryngismus, colic, hiccup, &c.; and cases have been detailed to me of its occasional successful use in asthma, spasmodic urethral stricture, &c. But there is one common and too fatal spasmodic disease, almost confined to the period of childhood, in which I have seen anæsthetic inhalations successful in arresting and controlling the paroxysms, and where probably a more extended and persevering use in the employment of them would be found to be attended with beneficial effects. I allude to hooping cough. I have known chloroform inhalations greatly abate the irritability of the cough attendant upon phthisis, &c. But with others I have scrupled to use chloroform inhalations in hooping-cough, under the fear that they might possibly increase the great predisposition which exists in this affection to pneumonic inflammation, or aggravate that inflammation if it were already present. This *a priori* reason, however, against the use of chloroform inhalations as an antispasmodic in hooping-cough has been of late set aside by the observations and experience of different German physicians. In a paper containing some remarks relative to the medical uses of chloroform, published in the *Monthly Journal* for December, 1847, in addition to its employment as an antispasmodic, anodyne, &c., I suggested the possibility of the drug acting as a contra-stimulant in some inflammatory diseases, and particularly those of a painful kind. Latterly we have had records published of its employment in upwards of 200 cases of pneumonia in German practice. Out of 193 cases of pneumonia treated with chloroform inhalations by Wachern, Baumgärtner, Helbing, and Schmidt, 9 patients died, or the mortality amounted to 4¼ per cent. Dr. Varrentrapp has given chloroform in 23 cases of pneumonia in the Frankfort Hospital. One of these 23 patients died. The detailed results in the other 22 cases seem to have been sufficiently satisfactory. At all events, the effects of the chloroform inhalations upon the cough, expectoration, &c., and upon the general course of the disease, would appear to show that we need have no fears of deleterious effects from it, as far as regarded the chance or existence of pulmonary inflammation; whatever advantages we may derive from it in relation to its prevention of that inflammatory state by allaying the cough, keeping the lungs in a relative state of quietude, and abating or restraining the succession of characteristic spasmodic attacks. I speak of course of the more severe cases of pertussis; for the milder forms of it require care merely rather than actual treatment. —*Braithwaite's Retrospect* and *Monthly Journal.*

